# Supplementary material for: A putative cap binding protein and the methyl phosphate capping enzyme Bin3/MePCE function in telomerase biogenesis
Source: Nat Commun. 2022 Feb 25;13:1067. doi: 10.1038/s41467-022-28545-9 (PMC8881624; doi:10.1038/s41467-022-28545-9)
Supplement: Supplementary file 1 — Supplementary Information [file 41467_2022_28545_MOESM1_ESM.pdf]

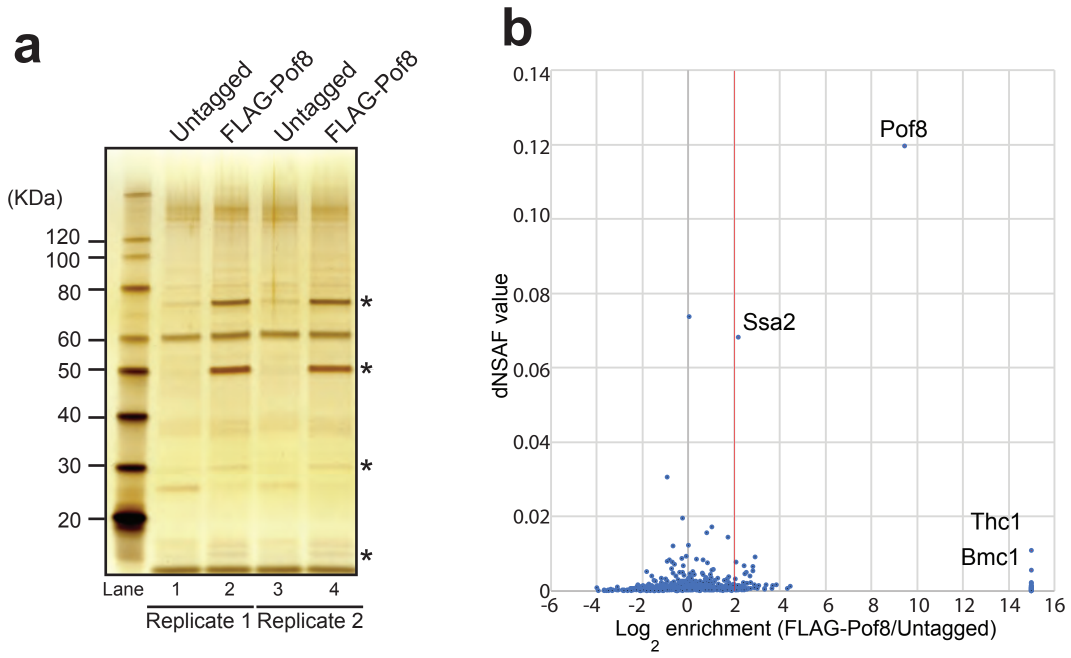

Supplementary Figure 1. Identification of Pof8 interacting proteins. **a** Silver stained SDS-PAGE of immunoprecipitations (IPs) for 3xFLAG-Pof8 expressed from a plasmid under the control of its endogenous promoter. The two bands marked with asterisks at ~50 kDa and ~75 kDa represent 3xFLAG-Pof8 and Ssa2, a heat shock protein, respectively. Two biological replicates are shown. Two lower bands are more prominent in lanes 2 and 4 compared to lanes 1 and 3 and are also marked with asterisks. **b** Scatter plot of proteins enriched in 3xFLAG-Pof8 IP by mass spectrometry. The x-axis shows the average enrichment in the 3xFLAG-Pof8 samples compared to untagged controls. Proteins not detected in the controls have a calculated enrichment value of infinity. To plot these data points, the enrichment value was arbitrarily set to 2^{15}. On the y-axis is the distributed Normalized Spectral Abundance Factor (dNSAF) mean value from tagged samples. The vertical red line marks a 4-fold enrichment in the tagged over untagged samples.

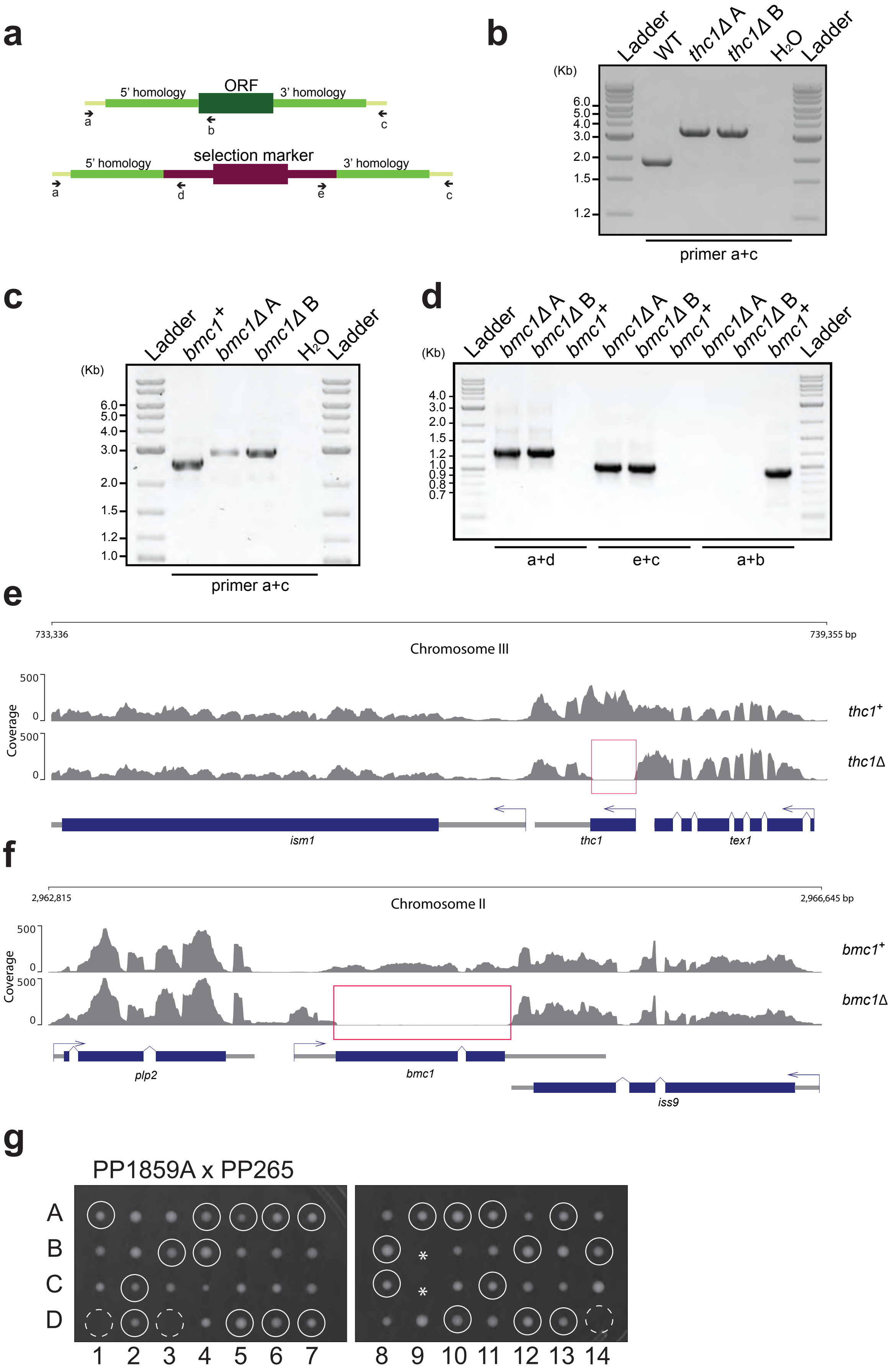
a
5' homology
ORF
3' homology
a
b
c
selection marker
d
e
b
Ladder
WT
thc1Δ A
thc1Δ B
H2O
(Kb)
6.0
5.0
4.0
3.0
2.0
1.5
1.2
primer a+c
c
bmc1+
bmc1Δ A
bmc1Δ B
1.0
d
a+d
e+c
a+b
0.9
0.8
0.7
e
733,336
739,355 bp
Chromosome III
500
0
Coverage
thc1+
thc1Δ
ism1
thc1
tex1
f
2,962,815
2,966,645 bp
Chromosome II
bmc1Δ
plp2
bmc1
iss9
g
PP1859A x PP265
A
B
C
D
1 2 3 4 5 6 7
8 9 10 11 12 13 14

Supplementary Figure 2. a Schematic of primers used to verify the deletion of *thc1* and *bmc1*, respectively. **b** Diagnostic PCR across the *thc1* locus using genomic DNA from two *thc1D* isolates and a *thc1*$^+$ control strain and primers a and c as indicated in the schematic in a. The lane labelled H_2O contained no genomic DNA. **c** Diagnostic PCR as in (b) but for the *bmc1* locus. **d** Diagnostic PCR to confirm the insertion of the *bmc1* knock-out cassette at the correct location in the genome. Primers a+d give a product of the expected size if insertion of the 5' homology occurred in the correct location; primers e+c give a product of the expected size if insertion of the 5' homology occurred in the correct location. Primers a+b only give a product if the *bmc1* open reading frame is present. **e** Coverage tracks from RNA-sequencing analysis for the *thc1* locus in a *thc1Δ* and *thc1*$^+$ control strain. The absence of reads mapping to the *thc1* open reading frame in the deletion strain boxed in red serves as an independent confirmation of the locus having been successfully deleted. **f** Coverage tracks from RNA sequencing analysis for the *bmc1* locus in a *bmc1Δ* and *bmc1*$^+$ control strain. **g** Tetrad dissection of a cross between *bmc1Δ* strain PP1859 and PP265 (wildtype *S. pombe* Lindner 972). Each column represents the spores from one tetrad. White circles denote *bmc1Δ* colonies based on growth following replica-plating to YEA NAT media. Dashed circles indicate *bmc1Δ* colonies that did not grow, the two asterisks in tetrad 9 label the positions of a *bmc1*$^+$ and a *bmc1Δ* spore that did not form colonies.

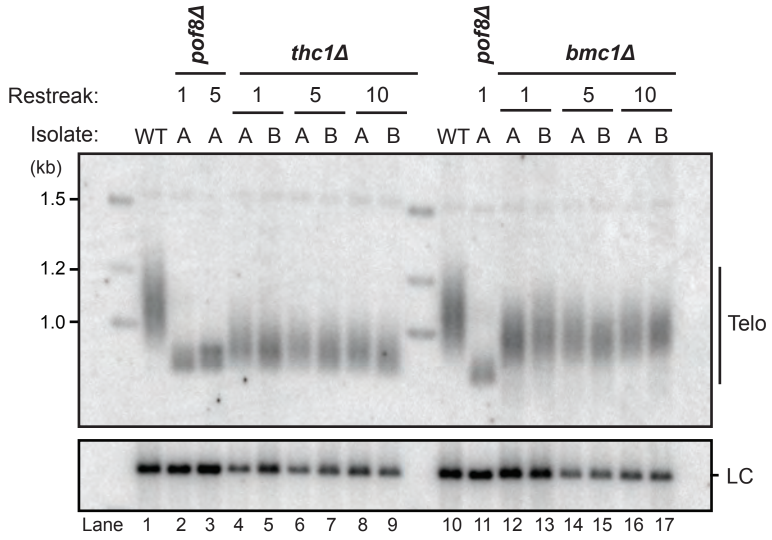

Supplementary Figure 3. Stable but short telomeres in thc1 and bmc1 deletion strains. Telomeric Southern blot comparing telomere length in wildtype (WT) and *thc1*Δ or *bmc1*Δ cells. Two independent isolates of each deletion (A and B) were restreaked ten times in series and telomere length was analyzed (one restreak equals 20-25 generations). A *pof8*Δ strain was included as control. The *rad16*$^{+}$ locus was probed to assess integrity and equal loading of the genomic DNA samples.

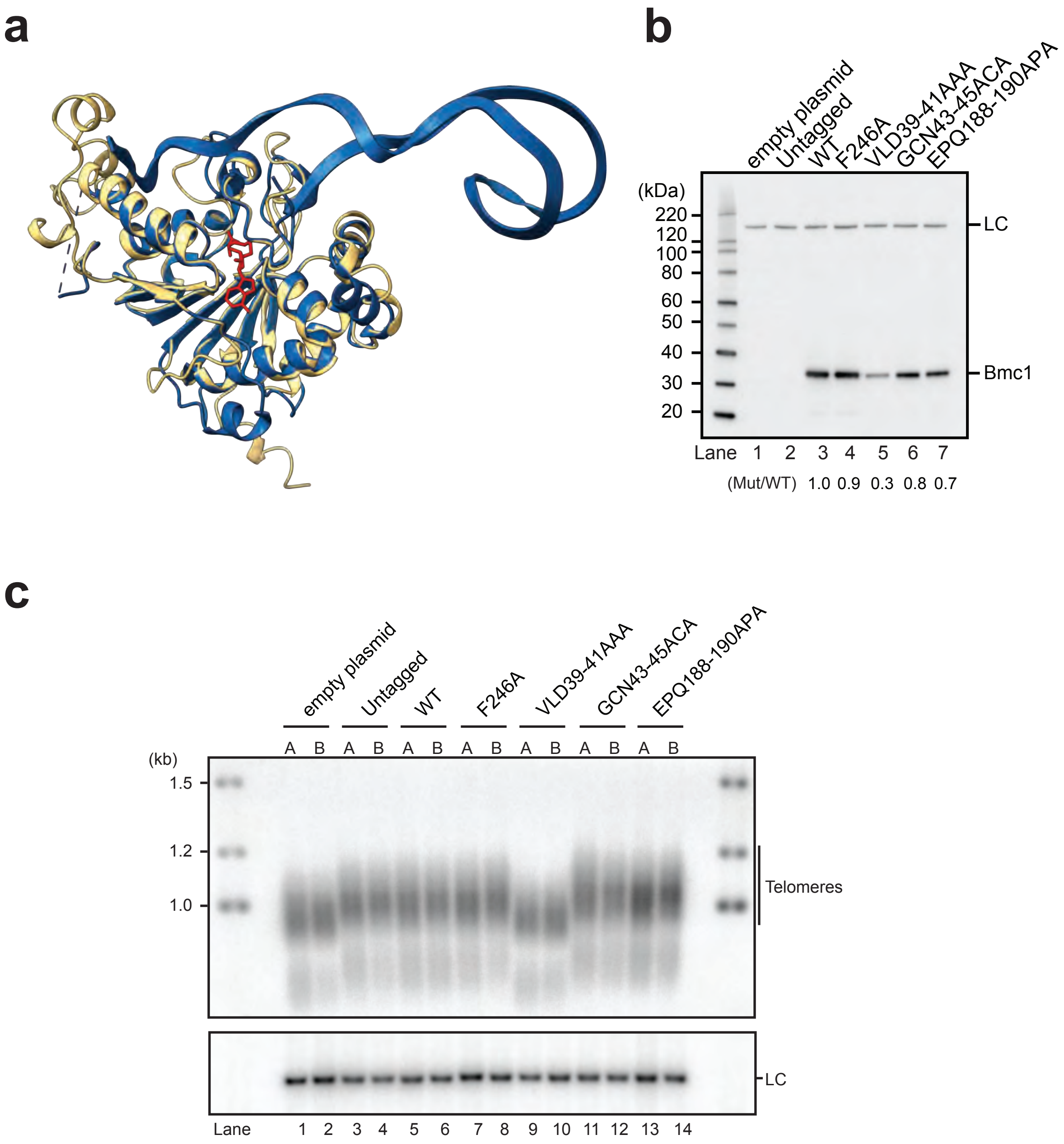

Supplementary Figure 4. The function of Bmc1 in telomerase biogenesis is independent of its catalytic activity. **a** ChimeraX-1.25 overlay of the Alphafold predicted structure of *S. pombe* Bmc1 (orange) and the human MePCE methyltransferase domain bound to S-adenosylhomocysteine (blue, PDB ID 6DCB). S-adenosylhomocysteine shown in red. **b** Western blot analysis of Twinstrep-tagged wildtype (WT) and mutant Bmc1 designed to affect the methyltransferase activity using α-Strep-tag II antibody. All versions of Bmc1 were expressed from plasmids under the control of the endogenous promoter in a *bmc1*Δ background. A non-specific band recognized by α-Strep-tag II in the absence of the epitope-tag (lane 2) was used as intrinsic loading control (LC). Quantification of mutants compared with WT are shown below the lane numbers. **c** Telomeric Southern blot for *bmc1* mutants. Two independent isolates of each strain are shown. A probe against the *rad16*$^{+}$ locus was used as LC. Lane numbers are indicated below the blot.

Paez et al.; Supplementary Figure 5

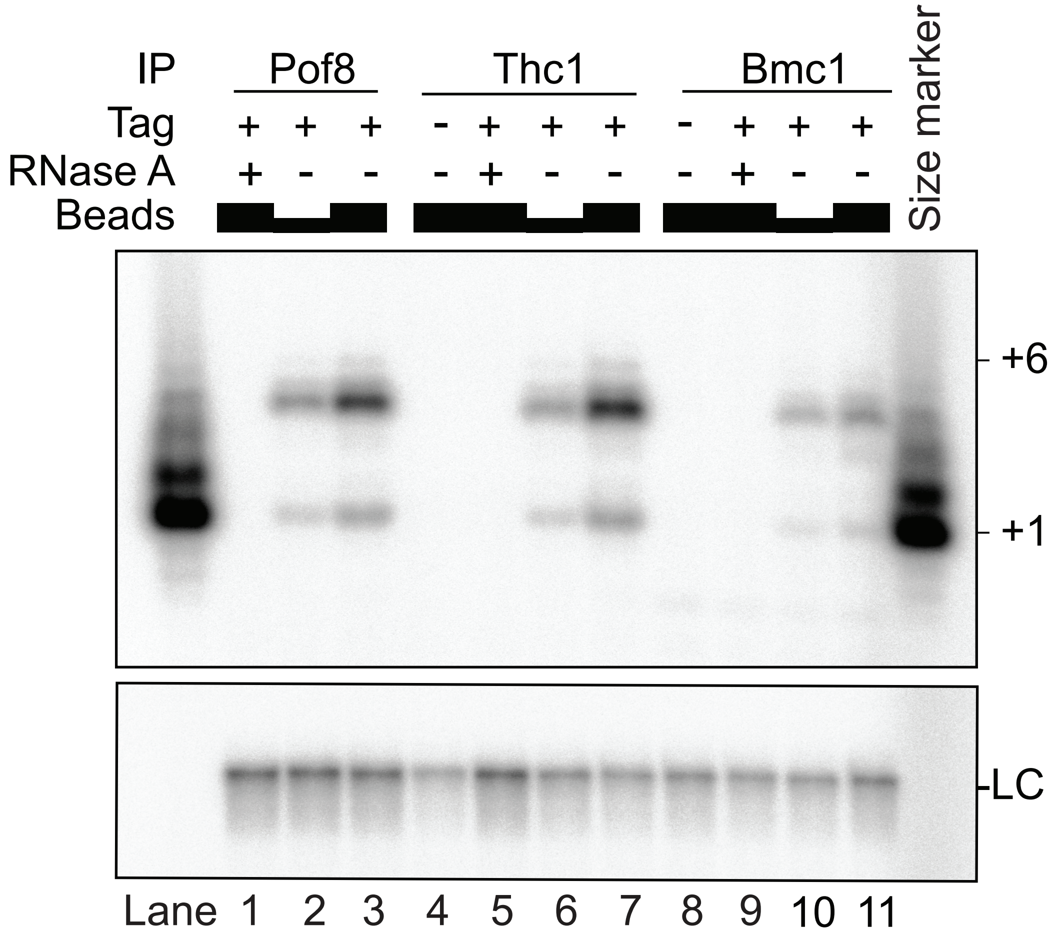

Supplementary Figure 5. Thc1 and Bmc1 are associated with telomerase activity. Activity assays were performed following immunoprecipation of 3xFLAG-Pof8 with anti-FLAG antibody coated Dynabeads, Thc1-2xV5 with anti-V5 antibody coated Dynabeads and Bmc1-Twinstrep with StrepTactin Sepharose. 10 µL (lanes 2, 6 and 10) or 20 µL (lanes 1, 3-5, 7-9 and 11) of IP suspensions were used for telomerase activity assay. A 32P labelled 100-mer oligo nucleotide was used as precipitation and loading control (LC).

Paez et al.; Supplementary Figure 6

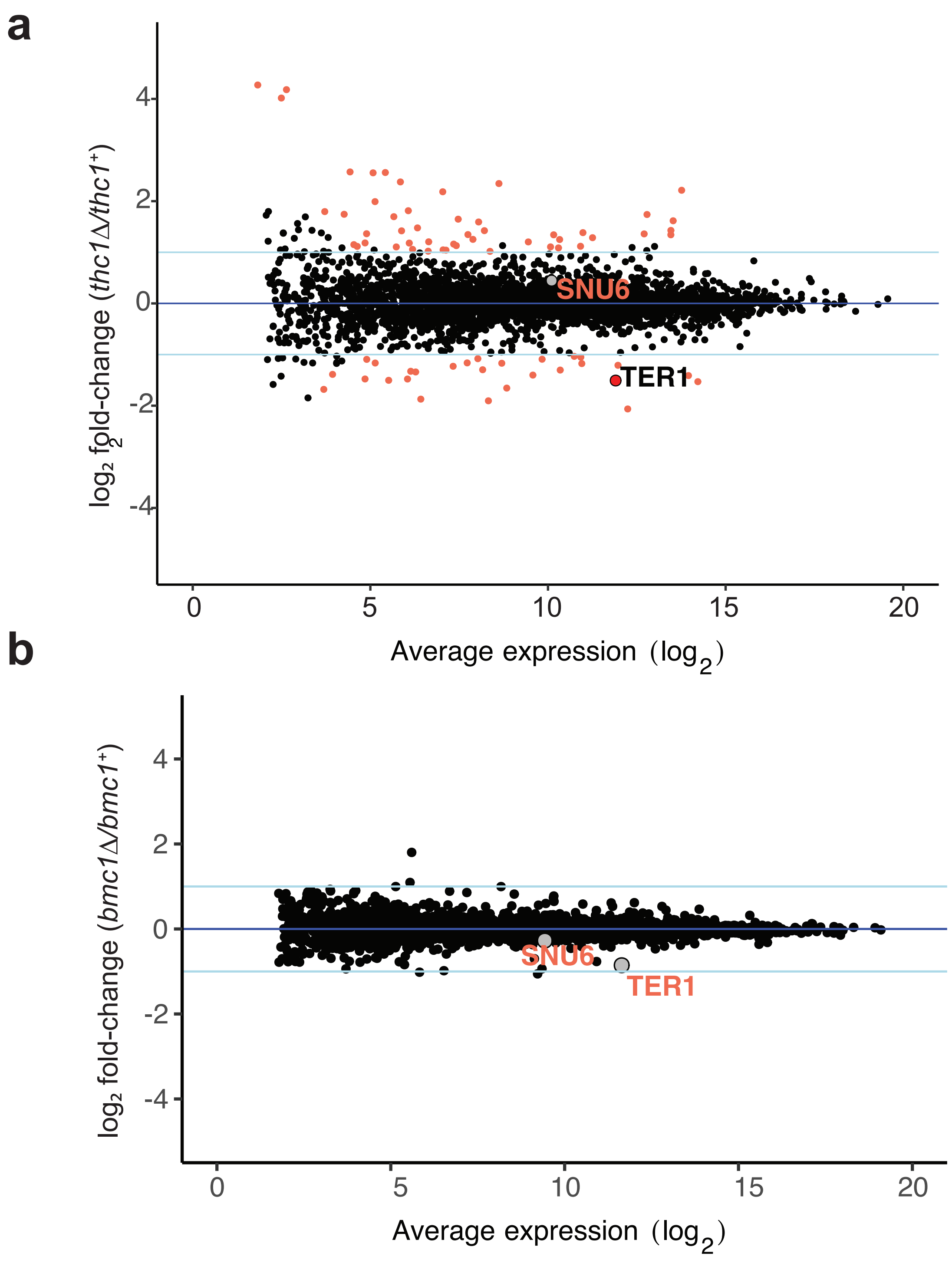

Supplementary Figure 6. MA plot of differential expression analysis for the deletion of *thc1* (**a**) and *bmc1* (**b**). Average expression is plotted on the x-axis and $\log_2$ fold change between the deletion and wildtype is plotted on the y-axis. Differentially expressed genes with an absolute $\log_2$ fold change of ≥ 1 and an adjusted p-value < 0.05 are colored in orange.

a

Figure 2a Figure 2b Figure 2c

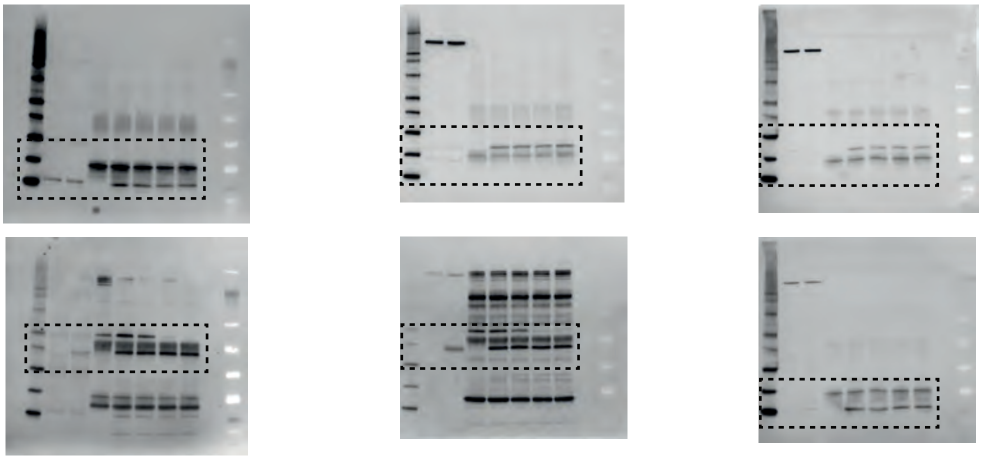

b

Figure 3d

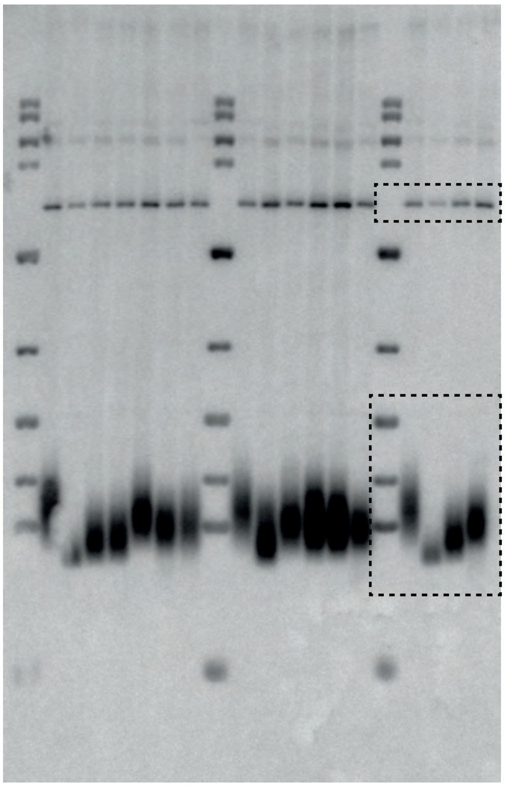

c

Figure 4b

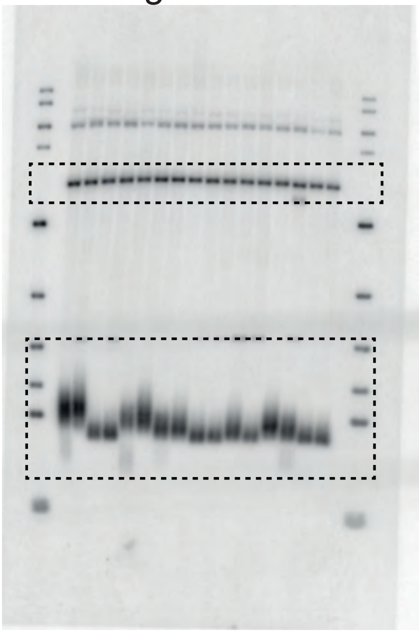

Figure 4c

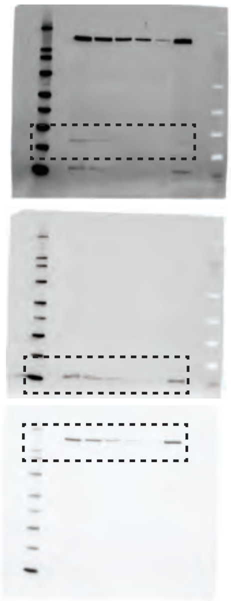

Figure 4d

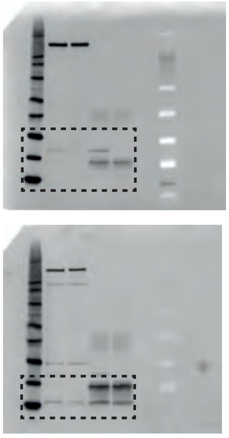

Figure 4e

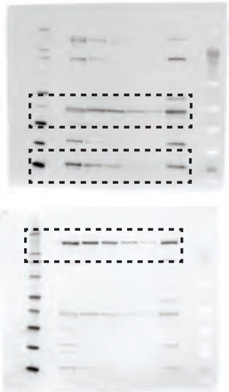

Figure 4f

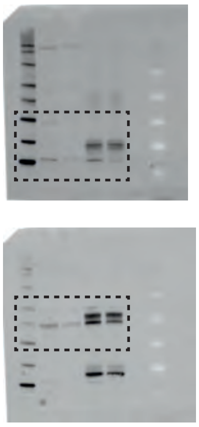

Figure 4g

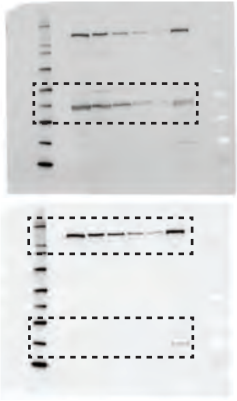

Figure 4h

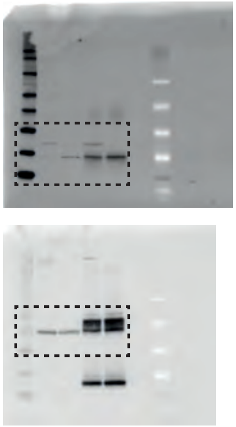

d

Figure 5a

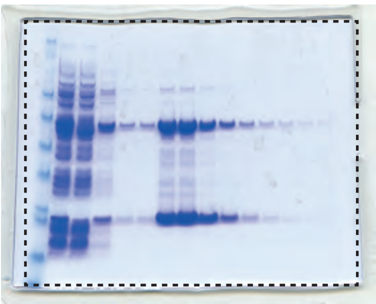

Figure 5b

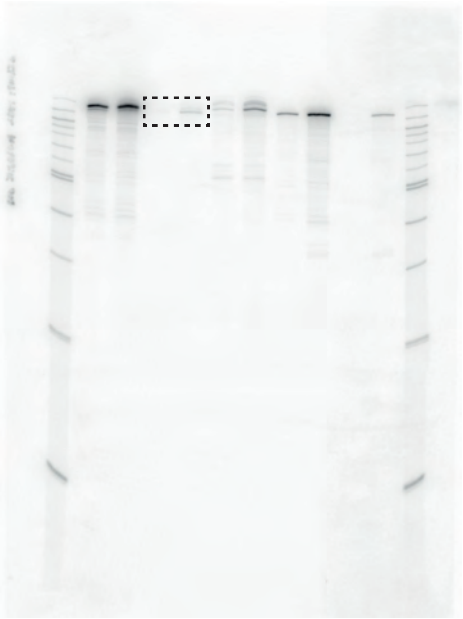

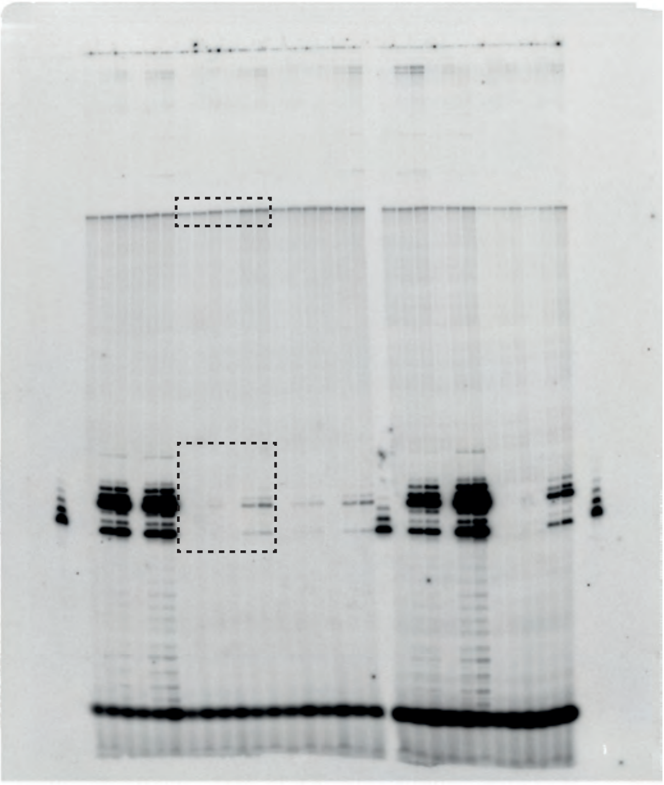

Figure 5c

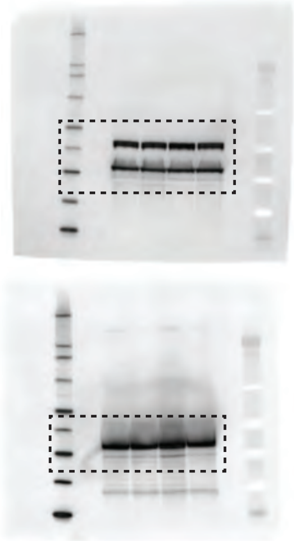

Figure 5e

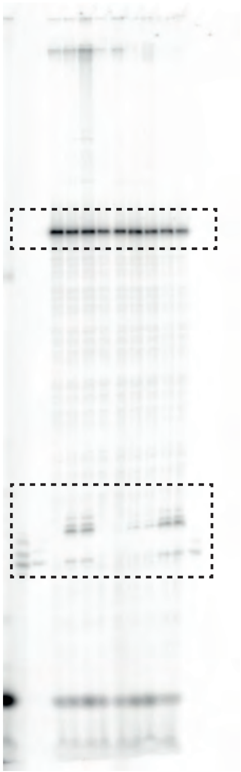

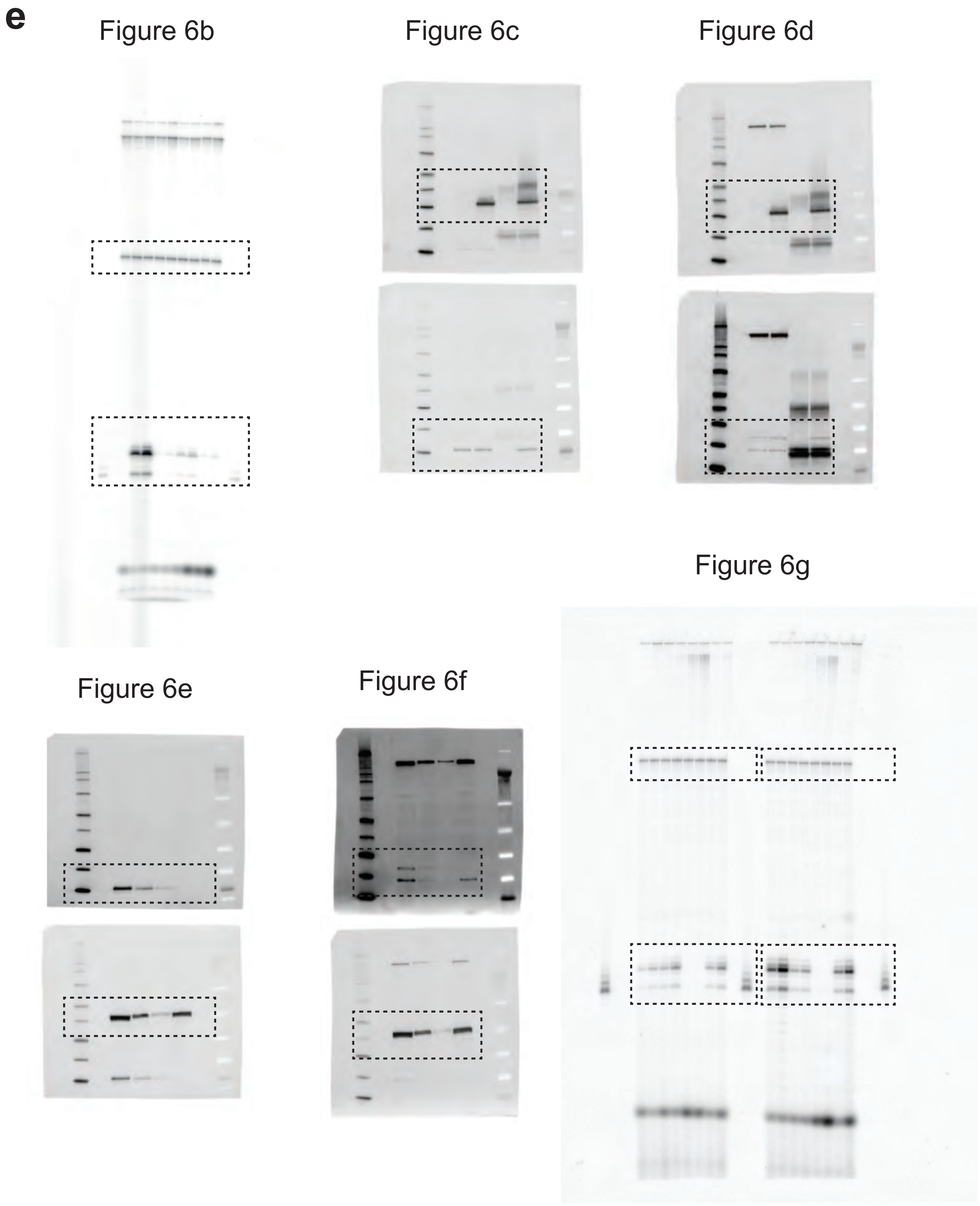

Supplementary Figure 7. Raw data presented in main figures. **a** Uncropped blots from Figure 2a-c. **b** Figure 3d. **c** Figure 4b-h. **d** Figure 5a-c, e. **e** Figure 6b-g.

Supplementary Table 1: Proteins enriched in Pof8-FLAG affinity purification with average dNSAF values >0.005 for the tagged Pof8 samples. Proteins are sorted by average fold enrichment in tagged Pof8 over untagged control.

NCBI_Gene	gene_ID	Description	Pof8 avg dNSAF	control avg dNSAF	log2 fold change Pof8/ctrl	MW (Da)
	SPCC18B5.09c	sequence orphan	0.010803	0	∞ (15)	13507
	SPBC2A9.10	Bin3 family, transcriptional and translational regulator (predicted)	0.005521	0	∞ (15)	30485
pof8	SPAC17G6.17	F-box protein Pof8	0.119549	0.000168	9.47	46808
leu1	SPBC1A4.02c	3-isopropylmalate dehydrogenase Leu1	0.008942	0.001156	2.95	39733
ubi5	SPAC589.10c	ubiquitin-40S ribosomal protein S31 fusion protein	0.006532	0.000904	2.85	17215
rvb2	SPBC83.08	AAA family ATPase Rvb2	0.005249	0.000729	2.85	51562
hsp90	SPAC926.04c	Hsp90 chaperone	0.006587	0.001186	2.47	80596
ssa2	SPCC1739.13	heat shock protein Ssa2 (predicted)	0.068151	0.014617	2.22	70233
mcp60	SPAC12G12.04	mitochondrial heat shock protein Hsp60/Mcp60	0.007729	0.001784	2.12	62168
rpp203	SPAC1071.08	60S acidic ribosomal protein P2C	0.014391	0.004215	1.77	11114
rps3	SPBC16G5.14c	40S ribosomal protein S3	0.005661	0.002627	1.11	27553
tdh1	SPBC32F12.11	glyceraldehyde-3-phosphate dehydrogenase Tdh1	0.005581	0.002685	1.06	35870
rpp201	SPBP8B7.06	60S acidic ribosomal protein P2A	0.017156	0.008308	1.05	11158
ssc1	SPAC664.11	mitochondrial heat shock protein Hsp70	0.00747	0.004123	0.86	72977
sks2	SPBC1709.05	heat shock protein, ribosome associated molecular chaperone Sks2	0.015436	0.008584	0.85	67206
	SPAC12G12.07c	conserved fungal protein	0.008371	0.006479	0.37	45724
lsm4	SPBC30D10.06	U6 snRNP-associated protein Lsm4 (predicted)	0.005178	0.004473	0.21	13941
cxr1	SPBC23E6.01c	mRNA processing factor (predicted)	0.07353	0.070509	0.06	51705
pabp	SPAC57A7.04c	mRNA export shuttling protein	0.012228	0.011967	0.03	71513

Supplementary Table 2: Genes affected in expression level by the deletion of *thc1*
a Genes with RNA levels decreased by more than 2-fold in the absence of *thc1*⁺ based on DESeq2 analysis of RNA samples from triplicate cultures of *thc1*⁺ and *thc1Δ* cells. A two-sided Wald test was used and adjusted *p*-values were calculated by Benjamini-Hochberg **b** Genes with increased expression by more than 2-fold by the same analysis as in (a).

a

gene_ID	external_gene_ID	log2_FC	adj. p-value	gene_biotype
SPNCRNA.863	SPNCRNA.863	-2.0640	9.6E-141	ncRNA
SPNCRNA.1344	SPBC1271.08c-antisense-1	-1.9046	6.7E-34	ncRNA
SPCC569.02c	SPCC569.02c	-1.8719	1.2E-12	protein_coding
SPBCPT2R1.07c	SPBCPT2R1.07c	-1.6810	2.5E-02	pseudogene
SPNCRNA.742	SPAC9.08c-antisense-1	-1.6559	3.5E-16	ncRNA
SPBP4G3.02	pho1	-1.5304	2.7E-236	protein_coding
SPNCRNA.214	ter1	-1.5083	7.8E-128	ncRNA
SPNCRNA.923	SPNCRNA.923	-1.5039	1.5E-05	ncRNA
SPNCRNA.130	omt3	-1.4787	4.7E-06	ncRNA
SPNCRNA.1255	SPNCRNA.1255	-1.4776	6.9E-04	ncRNA
SPAC821.10c	sod1	-1.4115	1.0E-138	protein_coding
SPCC70.08c	SPCC70.08c	-1.4017	1.2E-42	protein_coding
SPCC663.14c	trp663	-1.3878	4.1E-02	protein_coding
SPCC1393.14	ten1	-1.3405	5.2E-07	protein_coding
SPNCRNA.1605	SPNCRNA.1605	-1.3271	3.2E-07	ncRNA
SPCC18B5.09c	thc1	-1.3033	2.0E-76	protein_coding
SPNCRNA.888	end4-antisense-1	-1.2969	8.5E-23	ncRNA
SPAC186.06	SPAC186.06	-1.2292	5.1E-09	protein_coding
SPNCRNA.1626	SPNCRNA.1626	-1.2100	4.9E-44	ncRNA
SPBC1271.07c	SPBC1271.07c	-1.1767	4.9E-24	protein_coding
SPBPB2B2.01	SPBPB2B2.01	-1.1699	1.4E-24	protein_coding
SPBPJ4664.03	mfm3	-1.1656	4.3E-03	protein_coding
SPNCRNA.1340	SPNCRNA.1340	-1.1631	5.2E-15	ncRNA
SPAC8E11.12	SPAC8E11.12	-1.0941	1.5E-02	protein_coding
SPAC27D7.03c	mei2	-1.0916	3.0E-17	protein_coding
SPNCRNA.944	SPNCRNA.944	-1.0809	3.6E-05	ncRNA
SPBPB21E7.07	aes1	-1.0504	6.4E-25	protein_coding
SPAC1F7.08	fio1	-1.0350	3.4E-02	protein_coding

b

gene_ID	external_gene_ID	log2_FC	adj. p-value	gene_biotype
SPNCRNA.466	SPNG2151	4.2720	2.13E-02	ncRNA
SPAC977.05c	SPAC977.05c	4.1810	5.59E-03	protein_coding
SPBC1348.06c	SPBC1348.06c	4.0169	8.64E-03	protein_coding
SPMITTRNASER.01	SPMITTRNASER.01	2.5719	1.10E-05	tRNA
SPNCRNA.287	SPNCRNA.1300,SPNG1093	2.5602	2.39E-12	ncRNA

SPNCRNA.100	SPNCRNA.100	2.5543	7.41E-09	ncRNA
SPMITTRNALYS.01	SPMITTRNALYS.01	2.3763	1.32E-11	tRNA
SPNCRNA.1656	nup120-antisense-1	2.3445	1.38E-05	ncRNA
SPCC1393.10	ctr4	2.2131	3.19E-201	protein_coding
SPAC1F8.04c	SPAC1F8.04c	2.1839	3.42E-15	protein_coding
SPBP4H10.09	rsv1	1.9912	6.16E-04	protein_coding
SPNCRNA.1282	SPNCRNA.1282	1.8133	9.99E-11	ncRNA
SPNCRNA.1677	doa10-antisense-1	1.7962	8.50E-03	ncRNA
SPMITTRNAMET.01	SPMITTRNAMET.01	1.7436	1.54E-03	tRNA
SPCC1235.14	ght5	1.7393	8.95E-134	protein_coding
SPNCRNA.601	SPNCRNA.601	1.6964	1.15E-07	ncRNA
SPAC1F8.03c	str3	1.6478	2.32E-07	protein_coding
SPAC1142.05	ctr5	1.6164	3.39E-148	protein_coding
SPBPB2B2.05	SPBPB2B2.05	1.5930	1.59E-03	protein_coding
SPNCRNA.1558	qcr10-antisense-1	1.4771	4.57E-08	ncRNA
SPRRNA.01	21S_rRNA	1.4280	7.37E-12	rRNA
SPNCRNA.1307	SPBPB10D8.02c-antisense-1	1.4233	9.50E-19	ncRNA
SPNCRNA.945	SPNCRNA.945	1.4199	5.60E-07	ncRNA
SPAC1F8.06	pfl8	1.3844	2.63E-05	protein_coding
SPNCRNA.983	tsn1-antisense-1	1.3631	6.86E-03	ncRNA
SPRRNA.02	15S_rRNA	1.3599	1.52E-10	rRNA
SPBP4H10.10	rbd3	1.3464	1.10E-09	protein_coding
SPCC330.06c	pmp20	1.3422	2.45E-49	protein_coding
SPBC11B10.02c	his3	1.3419	2.23E-54	protein_coding
SPAC4G8.03c	puf5	1.2856	8.00E-49	protein_coding
SPBC1778.04	spo6	1.2527	2.95E-07	protein_coding
SPNCRNA.634	shk2-antisense-1	1.2495	5.43E-64	ncRNA
SPNCRNA.1557	lid2-antisense-1	1.2051	2.49E-03	ncRNA
SPAC56F8.15	SPAC56F8.15	1.2029	8.85E-28	protein_coding
SPNCRNA.1438	cdc2-antisense-1	1.1829	6.63E-03	ncRNA
SPBC1289.14	SPBC8E4.10c	1.1804	2.69E-03	protein_coding
SPNCRNA.1409	SPNCRNA.1409	1.1604	6.19E-07	ncRNA
SPNCRNA.33	prl33	1.1499	3.74E-02	ncRNA
SPCC1739.08c	SPCC1739.08c	1.1300	2.14E-04	protein_coding
SPBC1D7.02c	scr1	1.1176	2.29E-42	protein_coding
SPNCRNA.1330	SPNCRNA.1330	1.1154	4.05E-02	ncRNA
SPBC23G7.08c	rga7	1.1060	7.65E-27	protein_coding
SPNCRNA.892	tif211-antisense-1	1.1049	2.85E-04	ncRNA
SPNCRNA.953	SPNCRNA.953	1.0861	1.32E-35	ncRNA
SPNCRNA.1031	wsp1-antisense-1	1.0573	1.98E-04	ncRNA
SPNCRNA.875	gcv1-antisense-1	1.0473	2.35E-08	ncRNA
SPNCRNA.1378	cbp6-antisense-1	1.0417	6.87E-09	ncRNA
SPNCRNA.774	SPNCRNA.774	1.0215	1.55E-02	ncRNA

SPCC794.01c	gcd1	1.0186	1.95E-09	protein_coding

Supplementary Table 3: *S. pombe* strains used in this study

Strain	Genotype	Source	Figure
FP1546	h^- *ade6-M216 leu1-32 ura4-D18 his3-D1 lsm4::lsm4-myc13-natMX6 pof8::kanMX6 [pDBlet-Pof8]*	Páez-Moscoso et al 2018	S1a
FP1547	h^- *ade6-M216 leu1-32 ura4-D18 his3-D1 lsm4:: lsm4-myc13-natMX6 pof8::kanMX6 [pDBlet-3xFLAGPof8]*	Páez-Moscoso et al 2018	S1a
FP1913	$h^?$ *ade6-M21? leu1-32 ura4-D18 his3-D1 bmc1(SPBC2A9.10)::natMX6 [ura4, pDBlet]*	This study	S4b,c
FP1914	$h^?$ *ade6-M21? leu1-32 ura4-D18 his3-D1 bmc1(SPBC2A9.10)::natMX6 [pDBlet–bmc1 WT]*	This study	S4b,c
FP1915	$h^?$ *ade6-M21? leu1-32 ura4-D18 his3-D1 bmc1(SPBC2A9.10)::natMX6 [pDBlet–bmc1-Twinstrep]*	This study	S4b,c
FP1916	$h^?$ *ade6-M21? leu1-32 ura4-D18 his3-D1 bmc1(SPBC2A9.10)::natMX6 [pDBlet–bmc1-Twinstrep F246A]*	This study	S4b,c
FP1917	$h^?$ *ade6-M21? leu1-32 ura4-D18 his3-D1 bmc1(SPBC2A9.10)::natMX6 [pDBlet–bmc1-Twinstrep VLD39-41AAA]*	This study	S4b,c
FP1918	$h^?$ *ade6-M21? leu1-32 ura4-D18 his3-D1 bmc1(SPBC2A9.10)::natMX6 [pDBlet–bmc1-Twinstrep GCN43-45ACA]*	This study	S4b,c
FP1919	$h^?$ *ade6-M21? leu1-32 ura4-D18 his3-D1 bmc1(SPBC2A9.10)::natMX6 [pDBlet–bmc1-Twinstrep EPQ188-190APA]*	This study	S4b,c
PP137	h^+ *ade6-M216 leu1-32 ura4-D18 his3-D1*	Lab stock	3a, S2
PP138	h^- *ade6-M216 leu1-32 ura4-D18 his3-D1*	Lab stock	3a, 3b, 3c, 3d, S2b,c,d,e, S3, S5, S6
PP139	h^- *ade6-M210 leu1-32 ura4-D18 his3-D1*	Lab stock	3a, S2
PP265	h^-	ATCC	S2g
PP1723	h^- *ade6-M216 leu1-32 ura4-D18 his3-D1 pof8::kanMX6*	Páez-Moscoso et al 2018	3a, 3b, 3d
PP1797	$h^?$ *ade6-M21? leu1-32 ura4-D18 his3-D1 lsm4::lsm4-myc13-nat aur1::[pCST159-ter1] pof8::kanMX6*	This study	5b
PP1839	$h^?$ *ade6-M210/M216 ura4-D18 leu1-32 his3-D1 pof8::3xFLAG-pof8-kanMX6*	This study	3c, S5
PP1843	h^- *ade6-M216 leu1-32 ura4-D18 his3-D1 thc1 (SPCC18B5.09c):: thc1-TEV-2xV5-natMX6*	This study	2a, 3c, S5
PP1844	h^- *ade6-M216 leu1-32 ura4-D18 his3-D1 bmc1(SPBC2A9.10)::bmc1-TEV-TwinStrep-natMX6*	This study	2b, 3c, S5
PP1845	h^- *ade6-M216 ura4-D18 leu1-32 his3-D1 pof8::3xFLAG-pof8-kan thc1 (SPCC18B5.09c):: thc1-TEV-2xV5-natMX6*	This study	2a, 4e, 4f
PP1846	h^- *ade6-M216 ura4-D18 leu1-32 his3-D1 pof8::3xFLAG-pof8-kan bmc1(SPBC2A9.10)::bmc1-TEV-TwinStrep-natMX6*	This study	2b, 4g, 4h
PP1847	h^- *ade6-M216 leu1-32 ura4-D18 his3-D1 thc1 (SPCC18B5.09c)::his3*	This study	3a, 3b, 3d, S2b,e, S3, S6
PP1857	$h^?$ *ade6-M210/M216 leu1-32 ura4-D18 his3-D1*	This study	4a, 4b
PP1858	$h^?$ *ade6-M210 leu1-32 ura4-D18 his3-D1 pof8::KanMX6*	This study	4a, 4b
PP1859	h^+ *ade6-M210 leu1-32 ura4-D18 his3-D1 bmc1(SPBC2A9.10)::natMX6*	This study	4a, 4b, S2g
PP1860	$h^?$ *leu1-32 ura4-D18 his3-D1 ade6-M210/ade6-M216*	This study	3a, 3b, S2f, S3
PP1861	$h^?$ *leu1-32 ura4-D18 his3-D1 ade6-M210/ade6-M216 bmc1(SPBC2A9.10)::natMX6*	This study	3a, 3b, 3d, S2c,d,f, S3
PP1862	$h^?$ *ade6-M210 leu1-32 ura4-D18 his3-D1 thc1 (SPCC18B5.09c)::his3*	This study	4a, 4b
PP1863	$h^?$ *ade6-M210/216 leu1-32 ura4-D18 his3-D1 pof8::kanMX6 bmc1(SPBC2A9.10)::natMX6*	This study	4a, 4b
PP1864	$h^?$ *ade6-M216 leu1-32 ura4-D18 his3-D1 pof8::kanMX6 thc1 (SPCC18B5.09c)::his3*	This study	4a, 4b
PP1865	$h^?$ *ade6-M210/216 leu1-32 ura4-D18 his3-D1 bmc1(SPBC2A9.10)::natMX6 thc1 (SPCC18B5.09c)::his3*	This study	4a, 4b

PP1866	*h? ade6-M210 leu1-32 ura4-D18 his3-D1 pof8::kanMX6 bmc1(SPBC2A9.10)::natMX6 thc1 (SPCC18B5.09c)::his3*	This study	4a, 4b
PP1882	*h? ade6-M210 leu1-32 ura4-D18 his3-D1 smb1:: smb1-cMyc-natMX6, pof8::kanMX6*	This study	5f, 5g
PP1883	*h? ade6-M210 leu1-32 ura4-D18 his3-D1 smb1:: smb1-cMyc-natMX6, thc1 (SPCC18B5.09c)::his3*	This study	5f, 5g
PP1884	*h? ade6-M216 leu1-32 ura4-D18 his3-D1 smb1:: smb1-cMyc-natMX6, bmc1(SPBC2A9.10)::natMX6*	This study	5f, 5g
PP1885	*h? ade6-M210/M216 leu1-32 ura4-D18 his3-D1 smb1::smb1-cMyc-natMX6*	This study	5f, 5g
PP1886	*h? ade6-M216 leu1-32 ura4-D18 his3-D1 lsm4::lsm4-cMyc-natMX6, pof8::kanMX6*	This study	5c, 5d, 5e
PP1887	*h? ade6-M210 leu1-32 ura4-D18 his3-D1 lsm4::lsm4-cMyc-natMX6, thc1 (SPCC18B5.09c)::his3*	This study	5c, 5d, 5e
PP1888	*h? ade6-M216 leu1-32 ura4-D18 his3-D1 lsm4::lsm4-cMyc-natMX6, bmc1(SPBC2A9.10)::natMX6*	This study	5c, 5d, 5e
PP1889	*h? ade6-M210/M216 leu1-32 ura4-D18 his3-D1 lsm4::lsm4-cMyc-natMX6*	This study	5c, 5d, 5e
PP1892	*h? ade6-M216 leu1-32 ura4-D18 his3-D1 thc1 (SPCC18B5.09c)::thc1-TEV-2xV5-natMX6 bmc1(SPBC2A9.10)::bmc1-TEV-Twinstrep-natMX6*	This study	2c, 4c, 4d
PP1894	*h? ade6-M216 leu1-32 ura4-D18 his3-D1 bmc1(SPBC2A9.10)::bmc1-TEV-Twinstrep-natMX6*	This study	2c
PP1895	*h? ade6-M216 leu1-32 ura4-D18 his3-D1 pof8::kanMX6 thc1 (SPCC18B5.09c)::thc1-TEV-2xV5-natMX6 bmc1(SPBC2A9.10)::bmc1-TEV-Twinstrep-natMX6*	This study	4c, 4d
PP2014	*h? ade6-M21? leu1-32 his3-D1 ura4-D18 pof8::3xFLAG-pof8-kanMX6*	This study	6a, 6b
PP2015	*h? ade6-M21? leu1-32 his3-D1 ura4-D18 pof8::3xFLAG-pof8-kanMX6 thc1 (SPCC18B5.09c)::his3*	This study	6a, 6b
PP2016	*h? ade6-M21? leu1-32 his3-D1 ura4-D18 pof8::3xFLAG-pof8-kanMX6 bmc1(SPBC2A9.10)::natMX6*	This study	6a, 6b
PP2017	*h? ade6-M21? leu1-32 his3-D1 ura4-D18 pof8::3xFLAG-pof8-kanMX6 thc1 (SPCC18B5.09c)::his3 bmc1(SPBC2A9.10)::natMX6*	This study	6a, 6b
PP2024	*h? ade6-M21? ura4-D18 leu1-32 his3-D1 thc1::thc1-TEV-2xV5-natMX6 lsm4:: lsm4-cMyc-natMX6*	This study	6c, 6e, 6g
PP2025	*h? ade6-M216 ura4-D18 leu1-32 his3-D1 bmc1::bmc1-TEV-Twinstrep-natMX6 lsm4:: lsm4-cMyc-natMX6*	This study	6d, 6f, 6g
PP2038	*h? ade6-M21? ura4-D18 leu1-32 his3-D1 3xFLAG-pof8-kanMX6 thc1 (SPCC18B5.09c):: thc1-TEV-2xV5-natMX6 bmc1(SPBC2A9.10)::natMX6*	This study	4e, 4f
PP2039	*h? ade6-M21? ura4-D18 leu1-32 his3-D1 3xFLAG-pof8-kanMX6 bmc1(SPBC2A9.10)::bmc1-TEV-TwinStrep-natMX6 thc1(SPCC18B5.09c)::his3*	This study	4g, 4h

Supplementary Table 4: Oligonucleotides and gene synthesis products used to generate deletion, fusion and integration constructs

Product description	Primer #	Sequence
Nat MX6 for *bmc1* deletion	BLoli6066/BLoli2491	5'-TTTAGCTTGCCTCGTCCCCG-3'/5'-TGGATGGCGGCGTTAGTATC-3'
bmc1 5' homology	BLoli7736/BLoli7737	5'-ATCCTGAAGCGATGATGCCA-3'/ 5'-CGGGGACGAGGCAAGCTAAACCCAAGTCGAGGAGGTTTTT-3'
bmc1 3' homology	BLoli7738/BLoli7739	5'-GATACTAACGCCGCCATCCATTGTCTAGTAAAACGTTAAG-3'/5'-TTGGCGAGTATAACCAATGT-3'
bmc1::natMX6	BLoli7736/BLoli7739	5'-ATCCTGAAGCGATGATGCCA-3'/5'-TTGGCGAGTATAACCAATGT-3'
his3$^+$ for *thc1* deletion	BLoli7486/BLoli7487	5'-GTTTTGAAGACGGTGATACACGTTGTAATG-3'/5'-ATTTATCTGTTTGCTTATCGAACTATACGG-3'
thc1 5' homology	BLoli7484/BLoli7485	5'-CAATAATAACTTTGCTTACGATTAATAG-3'/5'-TGTATCACCGTCTTCAAAACTTTTGGTACC-3'
thc1 3' homology	BLoli7488/BLoli7489	5'CGATAAGCAAACAGATAAATTAGAACACAGC-3'/5'-GAGACTAATTGGGTTAAACAAAG-3'
thc1::his3	BLoli7484/BLoli7489	5'-CAATAATAACTTTGCTTACGATTAATAG-3'/5'-GAGACTAATTGGGTTAAACAAAG-3'
bmc1 5' homology for tagging	BLoli7718/BLoli7709	5'-AGCTCTCGAATTGGCCCTG-3'/5'-TTTAGAAGTGTTATTTCTCAAATTGAGGATGACTCCATG-3'
bmc1 3' homology for tagging	BLoli7541/BLoli7542	5'-GCCATCCAGTTAATTGTCTAGTAAAACGTTAAGAATAG/5'-TGATGTTGGCGAGTATAAC-3'
natMX6 cassette for *bmc1* tagging	BLoli7710/BLoli7540	5'-TGAGAAATAACACTTCTAAATAAGCGAATTTC-3'/5'-TAGACAATTAACTGGATGGCGGCGTTAG-3'
bmc1-Twinstrep::natMX6	BLoli7718/BLoli7542	5'-AGCTCTCGAATTGGCCCTG-3'/5'-TGATGTTGGCGAGTATAAC-3'
thc1 5' homology for tagging	BLoli7711/BLoli7712	5'-TCTTAAGATATTTGGGCTATAAATG-3'/5'-TTTAGAAGTGTTATTACGTGGAATCTAATCC-3'
thc1 3' homology for tagging	BLoli7715/BLoli7716	5'-GCCATCCAGTACAGATAAATTAGAACACAGC-3'/5'-AACAAAGTAGTAACCAAGG-3'
natMX6 cassette for *thc1* tagging	BLoli7713/BLoli7714	5'-CACGTAATAACACTTCTAAATAAGCGAATTTCTTATGATTTATG-3'/5'-ATTTATCTGTACTGGATGGCGGCGTTAG-3'
Thc1-2xV5::natMX6	BLoli7711/BLoli7716	5'-TCTTAAGATATTTGGGCTATAAATG-3'/5'-AACAAAGTAGTAACCAAGG-3'
3xFLAG-Pof8 5' homology for tagging	BLoli6676/BLoli6400	5'-AAAAGAATTCAACATGGCAACTGCGACCAA-3'/5'-TTTAGAAGTGTTACTTTTTAACATACGCCAATAATTC-3'
Pof8-kanMX6 3' homology for tagging	BLoli6401/BLoli6141	5'-GGCGTATGTTAAAAAAGTAACACTTCTAAATAAGCG-3'/5'-GCTTTCTTATTTGTAGAGACAATTG-3'
3xFLAG-Pof8-kanMX6	BLoli6676/BLoli6141	5'-AAAAGAATTCAACATGGCAACTGCGACCAA-3'/5'-GCTTTCTTATTTGTAGAGACAATTG-3'
Cloning of bmc1-Twinstrep into pDBlet	BLoli8069/BLoli8070	5'-CCGATAAGCTTAAACTATCTTAACCTGTCTACG-3'/5'-GGTGGCGGCCGCTACAGTTTTGGTATACCAGG-3'
bmc1 F246A 5' arm	Bloli8069/BLoli8006	5'-CCGATAAGCTTAAACTATCTTAACCTGTCTACG-3'/5'-GTTCGTTTGGCAGCATTCTTGTAC-3'
bmc1 F246A 3' arm	BLoli8007/BLoli8070	5'-GTACAAGAATGCTGCCAAACGAAC-3'/5'-GGTGGCGGCCGCTACAGTTTTGGTATACCAGG-3'
bmc1 VLD39–41AAA 5' arm	Bloli8069/BLoli7792	5'-CCGATAAGCTTAAACTATCTTAACCTGTCTACG-3'/5'-CATTATTGCATCCTATCGCTGCGGCTGAAGCCTC-3'
bmc1 VLD39–41AAA 3' arm	BLoli7793/BLoli8070	5'-CAGCCGCAGCGATAGGATGCAATAATGGGAC-3'/5'-GGTGGCGGCCGCTACAGTTTTGGTATACCAGG-3'
bmc1 GCN43–45ACA 5' arm	Bloli8069/BLoli7795	5'-CCGATAAGCTTAAACTATCTTAACCTGTCTACG-3'/5'-GAGCAGACACTGTCCCATTAGCGCACGCTATTGCT-3'
bmc1 GCN43–45ACA 3' arm	BLoli7796/BLoli8070	5'-GTGCGCTAATGGGACAGTGTCTGCTCAAATTG-3'/5'-GGTGGCGGCCGCTACAGTTTTGGTATACCAGG-3'
bmc1 EPQ188–190APA 5' arm	Bloli8069/BLoli7802	5'-CCGATAAGCTTAAACTATCTTAACCTGTCTACG-3'/5'-TCAAGTACGAGTCCCATCCGGCAGGTGCTAAAATAAG-3'
bmc1 EPQ188–190APA 3' arm	BLoli7803/BLoli8070	5'-ACCTGCCGGATGGGACTCGTACTTGAAAGCTG-3'/5'-GGTGGCGGCCGCTACAGTTTTGGTATACCAGG-3'
Bmp1 full length PCR verification	BLoli8048/BLoli7740	5'-GAGAGACTAGCACCAATGTATCC-3'/5'-TAGCTCTTGAAAAGGTACGG-3'

5' arm bmp1::natMX6 PCR verification	BLoli7735/BLoli3688	5'-CACTACAATTCAAGACTACC-3'/5'-GTAAGCCGTGTCGTCAAGAG-3'
3' arm bmp1::natMX6 PCR verification	BLoli7740/BLoli3791	5'-TAGCTCTTGAAAAGGTACGG-3'/5'-GGCGCTCTACATGAGCATGC-3'
5' arm bmp1 PCR verification	BLoli8048/BLoli8049	5'-GAGAGACTAGCACCAATGTATCC-3'/5'-AGGGAATCAGGCAAACATTTAAG-3'
thc1::his3 full length PCR verification	BLoli7484/BLoli7717	5'-CAATAATAACTTTGCTTACGATTAATAG-3'/5'-TGAAGCACCAAGATACGAGT-3'

Gene synthesis products

thc1-TEV-2xV5 fragment	BLgs1466	5'-TGCTGTATCGGAGCCTAATTCTAGTGGTCTTAAGATATTTGGGCTATAAATGATTGAAAATTGGCAATCTTGTTCACGCTGATCAACAATAAAAAAACGGCTACTTAGTATTTACCCTATACTCTTAGCATGGATACCTTCAATATTCATTGAAGTTGTAGTTTTAGCTTATTTGGTACCAAAAGTTTTGAAGAATGGAAGAGAAAAATACTGTTTCTTTATCTAAGCATATTGAACGTCCAGTAGAAGTTGTTGAAAGTCATTCTACGTACATTTTAAGTGCACAAGGACTTTATCTTACAGAACGCGTTTTAAGAAGTTATTTTAAACAACCTGATCTAATTATTACTTGGAAGGATAGTATGAGAGCTTACCTGACATTTTCTTCGCCGCAGGAAGCTCAAAAGGCTTACTTAGATTCACTTCGTTGGGGCAGTCAACTGAATGCTATCATTAAACCATTCTACGGTTCGCACGATGAGGTACTTCGTTTATGTAAAAGGAAAAGAATTATTCCACTACAAAATTTCTTGACTTCAGGTCTCGAGCCAACCACTGAGGATCTGTACTTTCAGAGCGATAACGATGGTAAGCCTATCCCTAACCCTCTCCTCGGTCTCGATTCTACGGGCGGAGGCTCCGGCGGCGGCTCTGGAGGATCTGCAGGTAAGCCAATACCCAACCCACTTCTTGGATTAGATTCCACGTAATAACACTTCTAAATAAGCGAAT-3'
bmc1-TEV-Twin-Streptag fragment	BLgs1465	5'-AGCTCTCGAATTGGCCCTGTACGCAATCCTGGTTCCATTGTAGAAGACCAGTTTAATTATTACCCCATTTCAAGCATTAAAAAGTTTTCCAGGATACCAGTGCAACTTCAACCACCTCTCAATAAGCAAAATTTCCCTCACAATATAGAATTTGAGACCGCTGACTTCTTGCGCTGGGAATCGAAACGAAAATTCAAAATAATACTAGCATTATCCGTATCTAAATGGGTGCATCTAAATAACCACGATGAAGGAATCATTAAATTCTTTGGGAAGATTAGTTCTTTATTGGAAACGAATGGTGTTCTTATTTTAGAACCTCAAGGATGGGACTCGTACTTGAAAGCTGCAAAAAAAAATATCTGTAAGTCACGACTATCAATACTTCTAACTCTTACTTTTCTAGGTTTTTAATCAAACACCTGAGAACCTCAAAATCCAACCTGATGCGTTTGAACATTTGCTTAATCAAGCAGGACTAGTGCTTGAATACAGTATCGAACCTCAAGTAAATAACTCTGAGTACAAGAATTTTGCCAAACGAACAATGTATATCTATAAAAAAAAAAGGAATTGGAATCATAAAACTATTAACTTCTACTCTCGAGCCAACAACTGAAGATTTATATTTTCAATCTGACAATGATTGGTCTCACCCACAGTTTGAAAAAGGCGGAGGCTCCGGCGGCGGCTCTGGAGGATCTGCATGGAGTCATCCTCAATTTGAGAAATAACACTTCTAAATAAGCGAATTTC-3'

Supplementary Table 5: Oligonucleotides used for RT-qPCR

**Product**	**Primer #**	**Sequence**	**Source**
TER1 all	BLoli7827	5'-CAGTGTACGTGAGTCTTCTGCCTT-3'	Ref. 15
	BLoli7828	5'-CAAAAATTCGTTGTGATCTGACAAGC-3'	Ref. 15
TER1 exon 2	BLoli7825	5'-AATTGCGTATTTAGTAAGAACGCG-3'	Ref. 16
	BLoli7826	5'-GATTCATCACTTTCTCAAAATTTTGAAACCG-3'	Ref. 16
act1	BLoli7829	5'-GGATTCCTACGTTGGTGATGA-3'	Ref. 16
	BLoli7830	5'-CGTTGTAGAAAGTGTGATGCC-3'	Ref. 16
his1	BLoli7818	5'-CGAAGACGTGCTTCAGCGA-3'	Ref. 15
	BLoli7819	5'-TGTCCACCTCGGAATCACTG-3'	Ref. 15
snR101	BLoli7866	5'-CGCTCTAGAAATTGGAATGAG-3'	This study
	BLoli7867	5'-TCTTAAAGGTGTGTCTCTCC-3'	This study
