# Supplementary material for: A putative cap binding protein and the methyl phosphate capping enzyme Bin3/MePCE function in telomerase biogenesis
Source: Nat Commun. 2022 Feb 25;13:1067. doi: 10.1038/s41467-022-28545-9 (PMC8881624; doi:10.1038/s41467-022-28545-9)
Supplement: Supplementary file 3 — Reporting Summary [file 41467_2022_28545_MOESM3_ESM.pdf]

Corresponding author(s): Peter Baumann
Last updated by author(s): 2021/05/11

Reporting Summary

Nature Research wishes to improve the reproducibility of the work that we publish. This form provides structure for consistency and transparency in reporting. For further information on Nature Research policies, see our Editorial Policies and the Editorial Policy Checklist.

Statistics

For all statistical analyses, confirm that the following items are present in the figure legend, table legend, main text, or Methods section.

n/a	Confirmed	
☐	☒	The exact sample size (*n*) for each experimental group/condition, given as a discrete number and unit of measurement
☐	☒	A statement on whether measurements were taken from distinct samples or whether the same sample was measured repeatedly
☐	☒	The statistical test(s) used AND whether they are one- or two-sided<br>*Only common tests should be described solely by name; describe more complex techniques in the Methods section.*
☒	☐	A description of all covariates tested
☐	☒	A description of any assumptions or corrections, such as tests of normality and adjustment for multiple comparisons
☐	☒	A full description of the statistical parameters including central tendency (e.g. means) or other basic estimates (e.g. regression coefficient) AND variation (e.g. standard deviation) or associated estimates of uncertainty (e.g. confidence intervals)
☐	☒	For null hypothesis testing, the test statistic (e.g. F, t, r) with confidence intervals, effect sizes, degrees of freedom and P value noted<br>*Give P values as exact values whenever suitable.*
☒	☐	For Bayesian analysis, information on the choice of priors and Markov chain Monte Carlo settings
☒	☐	For hierarchical and complex designs, identification of the appropriate level for tests and full reporting of outcomes
☒	☐	Estimates of effect sizes (e.g. Cohen's d, Pearson's r), indicating how they were calculated

Our web collection on statistics for biologists contains articles on many of the points above.

Software and code

Policy information about availability of computer code

Data collection	No software was used
Data analysis	fastx_toolkit (0.0.14), STAR (2.5.2b), SAMtools (1.9), DESeq2 (1.22.2), R (3.5), pyGenomeTracks (3.4), PMCMRplus (1.9.0), Microsoft Excel (v16.54), Image Lab 6.0.1, Design & Analysis Software 2.5.0, QuantStudio real-time PCR Software vl.3, (Fiji is Just) ImageJ vl.53c, ImageQuant TL (v7.0), Clustal Omega (1.2.4), HHpred (version: 57c8707149031cc9f8edceba362c71a3762bdbf8), AlphaFold (v. 2). Custom script used in this study are available at https://github.com/baumannlab/Sp_Paez_et_al_2021.

For manuscripts utilizing custom algorithms or software that are central to the research but not yet described in published literature, software must be made available to editors and reviewers. We strongly encourage code deposition in a community repository (e.g. GitHub). See the Nature Research guidelines for submitting code & software for further information.

Data

Policy information about availability of data

All manuscripts must include a data availability statement. This statement should provide the following information, where applicable:

- Accession codes, unique identifiers, or web links for publicly available datasets
- A list of figures that have associated raw data
- A description of any restrictions on data availability

The primary sequence data associated with this analysis has been deposited in NCBI's Gene Expression Omnibus (GEO) database under GEO accession number GSE174822. Raw mass spectrometry data and search results files have been deposited to the Proteome Xchange (http://proteomecentral.proteomexchange.org/cgi/GetDataset?ID=PXD026230) via the MassIVE repository and may be accessed via ftp://MSV000087493@massive.ucsd.edu with password "DJPM99055". Custom scripts used as part of this study are available at https://github.com/baumannlab/Sp_Paez_et_al_2021. Original data underlying this manuscript can be accessed from the Stowers Original Data Repository at http://www.stowers.org/research/publications/libpb-1664. Uncropped scans of gels and blots shown in Figs. 2a, b, c;

3d; 4b, c, d, e, f, g, h; 5a, b, c, e; 6b, c, d, e, f, g are also included in the Supplementary data (Suppl. Figure 7) associated with this manuscript. A source data file has been provided. A reporting summary for this Article is available as a Supplementary Information file. All reagents described in this study are available commercially or upon reasonable request from the authors.

Field-specific reporting

Please select the one below that is the best fit for your research. If you are not sure, read the appropriate sections before making your selection.

☒ Life sciences ☐ Behavioural & social sciences ☐ Ecological, evolutionary & environmental sciences

For a reference copy of the document with all sections, see nature.com/documents/nr-reporting-summary-flat.pdf

Life sciences study design

All studies must disclose on these points even when the disclosure is negative.

Sample size	No sample-size calculations were performed. For all the assays, sufficient numbers of technical and biological replicates were performed based on the observed biological and technical variability and according to prior experience and standard practices in the field.
Data exclusions	Data were only excluded for rare experiments when obvious technical problems prohibited data analysis (e.g. failed transfer of RNA from gel to membrane).
Replication	All experiments were successfully replicated. Generally, results are based on three independent experiments.
Randomization	We report on experiments where randomization is generally not required.
Blinding	Investigators were not blinded during experiments. Blinding was not necessary for studies of this type as phenotypes were objectively quantified (for example scored by algorithm) or the phenotype was either present or absent.

Reporting for specific materials, systems and methods

We require information from authors about some types of materials, experimental systems and methods used in many studies. Here, indicate whether each material, system or method listed is relevant to your study. If you are not sure if a list item applies to your research, read the appropriate section before selecting a response.

Materials & experimental systems

n/a	Involved in the study	
☐	☒	Antibodies
☒	☐	Eukaryotic cell lines
☒	☐	Palaeontology and archaeology
☒	☐	Animals and other organisms
☒	☐	Human research participants
☒	☐	Clinical data
☒	☐	Dual use research of concern

Methods

n/a	Involved in the study	
☒	☐	ChIP-seq
☒	☐	Flow cytometry
☒	☐	MRI-based neuroimaging

Antibodies

Antibodies used	As detailed in method section all antibodies were purchased and recognize well-characterized epitope tags. Here is the list of antibody used in this study: anti-c-Myc 9E10 (Sigma-Aldrich, M4439), anti-FLAG M2 (Sigma-Aldrich ,F3165), anti-V5 (Thermo fisher, R960-25), anti-strep-tag II (Abcam, ab76949), anti-cMyc (Santa Cruz Biotechnology, sc-789), anti-α-tubulin (Sigma, T5168), anti-V5 (Abcam, ab9116), anti-V5-HRP (Thermo fisher, R961-25), anti-mouse IgG (H+L) (Thermo Scientific, 31430), anti-rabbit IgG (H+L) (Thermo Scientific, 31460).
Validation	All antibodies were purchased and recognize well-characterized epitope tags as described by the manufactures' websites with the exception of anti-α-tubulin (Sigma, T5168) and are all validated by the manufactures. For antibodies recognizing well-characterized epitope tags, we further used untagged strains as controls to confirm identities of bands for the tagged proteins. anti-FLAG M2 (Sigma-Aldrich ,F3165) verification in Figure 2a, anti-V5 (Abcam, ab9116) verification in Figure 2c, anti-cMyc (Santa Cruz Biotechnology, sc-789) verification in Figure 6c, anti-strep-tag II (Abcam, ab76949) verification in Suppl. Figure 4b, anti-c-Myc 9E10 (Sigma-Aldrich, M4439) was previously verified on (https://doi.org/10.1038/nature10924). Validation information for anti-V5 (Thermo fisher, R960-25), anti-V5-HRP (Thermo fisher, R961-25) and anti-α-tubulin (Sigma, T5168) can be found on manufacturers website at https://www.thermofisher.com/antibody/product/V5-Tag-Antibody-Monoclonal/R960-25, https://www.thermofisher.com/antibody/product/V5-Tag-Antibody-Monoclonal/R961-25 and https://www.sigmaaldrich.com/DE/en/product/sigma/t5168?context=product, respectively.
